# Anthropo-toxicologia

**Published:** 1848

**Authors:** C. E. Lavender

**Affiliations:** Salem, Alabama


### ARTICLE V.

*Anthropo-toxicologia. Cases; with remarks. Read before the Alabama Medical Society, April 3d,* 1848. By C. E. Lavender, M.D. of Salem Alabama.

Not being able to find, in the works of Nosologis s, a name which conveys what I conceive to be the cause and pathological nature of the following cases, I am forced to adopt, or rather to coin from Greek, a word somewhat in accordance with my views. If I rightly apprehend the nature of these cases, my limited reading does not enable me to remember any recorded cases similer to them. Nor have I been able to find any allusion to a pathological condition, which I have supposed to exist in these cases. I make these remarks at the risk of being written down a very limited reader. I incur the risk however, with the hope of being enlightened on the subject; and for the purpose of excusing myself for the apparent pedantry of employing, in this advanced age, a new term in medicine.

The term *Anthropo-toxicologia*, it will be seen, is a Greek derivative, from *Anthropos*, man, and *toxicon*, poison, and it is intended to convey an idea of that form of *poison* generated in, or secreted from, one healthy person capable of producing disease in another human being subject to its influence.

That the human body in a state of disease is capable of sending forth a contagious or infectious poison, is familiar to every one. That the natural and normal secretions of certain animals are poisonous, and will, when received in the human system, produce disease and death, is equally true. But that the secretions or exhalations from some human beings in health, are so virulent and noxious as to cause disease in other healthy persons, is a position that will not readily be conceded, and must, therefore, be examined. For the purpose of throwing light upon this subject, I offer the following cases, which I think will be found interesting, whatever theory may be employed in explaining them.

Case I. Mrs. E. K. æt. 25, good constitution, mixed temperament, black hair and eyes, rosy cheeks, rather full habit, cheerful temper, uninterrupted health, was married to P. K., early in 1845. Soon became pregnant, and began to decline; became pale and hydropic; in a few months abortion followed; Bad health continued; some improvement, and in the fall of 1845, again became *enciente.* Temperament now leucophlegmatic. Various diuretics and tonics used with but little benefit.

Feb. 11th, 1846. Delivered of a still-born child by a premature birth. Nothing extraordinary attended or followed parturition, save the fact that no red blood followed the cutting of the chord or the removal of the placenta. Quite exhausted, but cheerful. R. Nutritive diet, elder wine, laxats., chalybeates.

15th. Found her cheerful, complaining of little pain, but much oppression, and at times drowsiness; but little derangement of secretions; pulse 110, feeble, compressible, and undulating; sighs often and turns from side to side; deadly paleness and slight swelling about the eyes; tongue clean, but pale and colorless; little appetite, no nausea; roaring and uneasy feeling in the head, but no pain; light somewhat unpleasant to the eyes; *light and all white objects appear yellow; indistinctness of vision.* R. Wine, veg. bitters, tr. muriat. ferri, B. mass. pul. dover.

16th. Pulse more frequent, compressible and intermitting; more oppression, vision indistinct; *light looks red, white curtains appear red, dark objects yellow, or dull red;* pupils dilated. Complains of no pain, but is more restless, and desires to be turned oftener. R. Op. camph. wine, min. acids, light mercurials, and counter-irritants.

17th. Has sunk rapidly since yesterday. Nothing that she has taken has had any visible effect. *Light as red as scarlet;* some wandering of intellect; breathing more laborous. Sunk gradually and without complaint till she expired.

CASE II. Mrs. S. K., second wife of this same P. K., æt. circ. 25, sang. nerv. temperament, good health, sound constitution; accustomed to manual labor; married in July, 1846, became pregnant and began to decline. Took sundry patent nostrums, which caused further prostration. In about eight months hydropic symptoms supervened.

April 10th. Delivered of a living male child. No attendant circumstance worthy of remark. Directed magnesia, nourishing diet, etc.

17th. Found her debilitated and oppressed; no acute pain, but dull aching in the head; hydropic symptoms; appetite moderate; secretions scanty, otherwise normal; pulse 120, small and deep seated; light unpleasant to the eyes; some darkness and obscurity of vision.

R. Ars. sol. chalybeates, blue mass, wine, nourishing diet.

18th. Frequent and distressing attacks of dyspnœa, approaching syncope; otherwise as before. R. Pill. assafœt. every six hours, alternate with blue pill; wine occasionally.

19th. Attacks of dyspnœa frequent and distressing. Becomes cold occasionally, with feeble pulse, but no reaction; no action on the bowels; lochial discharge free; roaring in ears and optic illusions; *can see but one half of an object, and that looks green;* light somewhat painful to the eye; stupid, and somewhat comatose. R. Nit. acid, b. mass, quin. morph., wine, arrow root, sac, etc.

20th. No improvement in any of the symptoms. Can see but half of a person's face, *which looks green, with red spots;* pulse, as before, 120, feeble, intermitting. R. Camph. t. op., t. stram., b. mass, wine; dry mustard to spine, blister to neck.

21st. No improvement; rather comatose; no complaint of head; *light and white objects red;* pulse 120; complains of the mouth. R. T. stram. 6 gla., ter die, wine, panada.

22d. But little alteration; rests well. Continued treatment.

23d, A. M. Healthy bilious stool; more in her senses; rests well. At 4 P. M., an exceedingly offensive watery purging came on, which caused great prostration; moderated in a few hours by the use of sac. sat. ap. and astgt. enemat. R. Sinapisms, burnt brandy, camph., ammonia, sac. sat., tr. opi.

24th. Purging moderated, but all the symptoms worse Comatose, with optic illusions. Secretions from the skin, etc., so offensive as to require purifiers; pulse 120, thread-like; cold extremities; apparently moribund. R. Quin. grs. 20; tr. opi. gtt. 20; to be repeated in six hours.

25th. Has had several cold spells, with threatened spasms; smell not so offensive, still necessary to keep something burning in the room; blisters on legs and back filled with yellow water. R. Quin. gr. 10; g. camph. gr. 2; morph. gr. $\frac{1}{8}$; every six hours.

26th. Symptoms as before; no improvement. Continue treatment.

27th. Rests quietly; some light delirium; offensive smell measurably removed; takes panada, etc. Continue treatment.

28th. More in her senses; still cadaverous look and optic illusions; petechiæ and blisters continue. Complains of quinine affecting her head. No action on bowels for 48 hours. R. Nit. acid, A. M.; magnesia, P. M.; morph. at night.

29th. Found her narcotized. Cr. tr. and sulph. dad been given last night, without my knowledge, which brought on excessive purging; a large amount of opiates had been given to check it. The patient gradually sunk, and died next morning.

Case III. Mrs. W.—This case, in its rise, progress, diagnostic symptoms, and result, entirely similar to the foregoing.

*Remarks.*—The toxicological symptoms in these cases are the constant and gradual emaciation and waste of vitality, without any apparent fixed disease; the optic illusions, especially the fact of *light appearing scarlet, and white red;* the coincidence of the symptoms under similar circumstances; and the fatal termination.

The anthropological facts are these: P. K., the husband of the two women whose cases are detailed (and the remarks will equally apply to D. W., the husband of the third,) is a man whose cutaneous secretions, especially under excitement, are known to be exceedingly offensive and disgusting. "Essentially an unclean animal, all the civet of the apothecary, mixed with the perfumes of Araby the blessed, cannot sweeten him." Is it not possible—nay, is it not probable, that the inhalation of these gaseous secretions or the absorptions of others, from this living laboratory of malaria, may have produced deleterious impressions on those constantly subjected to their influence. causing a specific and fatal train of morbid associations?

These two women were in the prime of life, in robust health, of good constitution, and of active, healthful habits. So soon as they came in contact with this man, they began to exhibit the effects of morbific causes. They both became leucophlegmatic, and began constantly and steadily to decline. The same optic illusions, dimness of vision, and perversion of colors marked the progress of both cases to a fatal termination.

The matter of contagion is no more cognizable to our senses than malaria. Infectious disease is, however, usually attended by a certain very sensible fœtor. Persons, whose bodies, in a state of health, are capable of elaborating infection, as far as my observation extends, are distinguished by the strong and peculiarly offensive character of their pulmonary and cutaneous exhalations. Although this may not always be the case, yet so signally is it true in the cases before us, that I have heard sensible men say that, in their opinion, it would cause disease in any person who would room with them. I, therefore, set this morbific agent down in the catalogue of animal poisons, as human infection, *sui generis.* What is its nature, its essence—from what part of the body derived, and upon what tissue it first makes its attack, are at present matters of conjecture. Whether all persons are obnoxious to this poison, or whether there is a peculiarity in some, rendering them more susceptible of its action than others, time and observation must decide. In the cases above noted the noxious cause evinced its power by depressing the vital energies, impoverishing the vital fluid, and deranging the sensorium. There are, doubtless, instances in which the infection, not being sufficiently concentrated or virulent to cause fatal consequences, makes known its existence in the continued bad health and low spirits of its victim. And this, it may be, is the secret why so many hysterical cases are so difficult to be controlled, and why, in such cases, the experience of our profession finds it profitable to prescribe temporary *absque marito.*

I have brought these cases to the notice of the Society, in order to call attention to this peculiarity in the human structure, and to this form of disease. When the first case presented itself to me, I looked upon it as a case of hydropic affection with *ramollisement* of the brain. When the next case occurred with all the peculiarities of the first, I very naturally suspected a similarity of cause. No other known cause presenting itself, I have deemed the one named sufficient to explain the phenomena. The third case, similar in all respects, both as regards cause and disease, strengthened my conviction. Nor is this conclusion, in my judgement, either rash or unreasonable. Personal cleanliness is conducive to health---the reverse, predisposes to disease. Healthy persons crowded together in a tight room, or in the hold of a ship, will suffer and contract disease from exposure to their own secretions and exhalations. Writers on hygiene, say it is hurtful to the health of the very young to sleep with the very old. It is not, therefore, more true that "evil communications corrupt good manners," than it is that evil bodily associations impair the health and superinduce disease.

---

REMARKS.---The above cases are both curious and interesting, and we are disposed to think Dr. Lavender correct in ascribing the gradual decline and ultimate death of two of his cases. to the fœtid and filthy exhalations arising from the body of their husband. The subject deserves consideration, and we hope the publication of these cases will excite the attention of physicians and elicit some further remarks on this curious question.

Whilst on this subject, it may not be out of place to allude to a singular prejudice which exists in Havana among those born of Spanish and native parents. Into the private and public hospitals of that city, nothing can induce a native of that Island to enter as a patient, if its known that one or more cases of Phthisis are quartered in the institution; yet at the same time, they neither fear the yellow fever nor any other form of febrile disease, although liable to contract such diseases. They argue that the exhalations, secretions, etc., etc,, from the consumptive not only predispose those confined in the same room to the same affection, but also deteriorate their general health, impair the tone and vigor of their constitutions, and thus prevent their speedy convalescence from other diseases. Popular prejudice sometimes has truth for its foundation, and such may be the case in this instance.

We regret that no post-mortem examination was made in these cases, as without this test they must be unsatisfactory and incomplete.—*N. Orleans Med. & Surg. Jour.*

